# Integrated Analysis of RNA-Binding Proteins Associated With the Prognosis and Immunosuppression in Squamous Cell Carcinoma of Head and Neck

**DOI:** 10.3389/fgene.2020.571403

**Published:** 2021-01-11

**Authors:** Guangsheng Hu, Qingshan Jiang, Lijun Liu, Hong Peng, Yaya Wang, Shuyan Li, Yanhua Tang, Jing Yu, Jing Yang, Zhifeng Liu

**Affiliations:** ^1^Department of Gastroenterology, The First Affiliated Hospital of University of South China, Hengyang, China; ^2^Department of Otorhinolaryngology, The First Affiliated Hospital of University of South China, Hengyang, China; ^3^Hunan Province Key Laboratory of Tumor Cellular & Molecular Pathology, Cancer Research Institute, University of South China, Hengyang, China

**Keywords:** squamous cell carcinoma of head and neck, RNA binding proteins, differentially expressed genes, prognosis, tumor immunity

## Abstract

RNA-binding proteins (RBPs) interacting with target RNAs play essential roles in RNA metabolism at the post-transcription level. Perturbations of RBPs can accelerate cancer development and cause dysregulation of the immune cell function and activity leading to evade immune destruction of cancer cells. However, few studies have systematically analyzed the potential prognostic value and functions of RBPs in squamous cell carcinoma of head and neck (SCCHN). Here, for the first time, we comprehensively identified 92 differentially expressed RBPs from The Cancer Genome Atlas (TCGA) database. In the training set, a prognosis risk model was constructed with six RBPs, including NCBP2, MKRN3, MRPL47, AZGP1, IGF2BP2, and EZH2, and validated by the TCGA test set, the TCGA all set, and the GEO data set. In addition, the risk score was related to the clinical stage, T classification, and N classification. Furthermore, the high-risk score was significantly correlated with immunosuppression, and low expression of EZH2 and AZGP1 and high expression of IGF2BP2 were the main factors. Thus, the risk model may serve as a prognostic signature and offer highlights for individualized immunotherapy in SCCHN patients.

## Introduction

Globally, squamous cell carcinoma of head and neck (SCCHN) represents the sixth most common malignancy, with increasing incidence and over 300,000 deaths annually (Ferlay et al., 2015; Siegel et al., 2020). The major causes of SCCHN include alcohol consumption, tobacco use, and human papilloma virus (HPV) infection (Hill and D’Andrea, 2019). Despite advances in multimodal treatments, including surgery, chemotherapy, and radiotherapy, the 5-year survival rate has not notably improved (Cramer et al., 2019). Hence, new reliable and prospective biomarkers are urgently required for efficient diagnosis and prognosis assessment and the development of therapeutic strategies to decrease the mortality rates of SCCHN patients.

RNA-binding proteins (RBPs) serve as post-transcriptional regulators interacting with target RNAs. Because RBPs play essential roles in RNA stability, alternative splicing, modification, translation, translation, and degradation, they impact the function and destiny of transcripts in the cell and maintain cellular homeostasis (Anantharaman et al., 2002; Mitchell and Parker, 2014; Pereira et al., 2017). Previous studies have shown that dysfunction of RBPs can eventually lead to multiple diseases ranging from hereditary diseases to cancers (Chelly and Mandel, 2001; Chénard and Richard, 2008; Neelamraju et al., 2018). In all, 1542 human RBPs, accounting for 7.5% of all protein-coding genes, interacting with all known RNA types have been identified utilizing deep-sequencing approaches (Gerstberger et al., 2014; Beckmann et al., 2015), which provide a rare opportunity for systematic analysis of RBP genes in cancers. However, few studies have comprehensively analyzed the relationship between RBPs and the prognosis of squamous cell carcinoma of head and neck (SCCHN).

Here, to comprehensively analyze the prognostic value and potential function of RBPs in SCCHN, we obtained gene expression profiles of SCCHN patients from The Cancer Genome Atlas (TCGA) database to construct a prognosis risk model. Interestingly, our study showed that the high-risk score was associated with immunosuppression.

## Materials and Methods

### Data Sets

The RNA sequencing (RNA-Seq) data and clinical information were downloaded from the TCGA database^1^ of 500 SCCHN patients with 44 adjacent normal samples. For TCGA data, we selected 498 SCCHN patients with follow-up data and randomly divided them into two groups: the TCGA training set (*n* = 298, Supplementary Table 1) and the TCGA test set (*n* = 298, Supplementary Table 2). Additionally, the GSE65858 data set was downloaded from the Gene Expression Omnibus (GEO) database^2^, as an external independent verification set with RNA-Seq data and clinic information. We performed data analysis utilizing R project (version 3.6.3)^3^. Clinical features of HNSCC patients of TCGA and GEO databases were shown in Table 1.

**TABLE 1:** Clinical characteristics of SCCHN patients in TCGA and GEO data sets.

Clinical characteristics	TCGA		GEO (GSE65858)	
	*n* = 499	%	*n* = 270	%
**Age**				
<60	219	43.9	153	56.7
≥60	280	56.1	117	43.3
**Gender**				
Female	132	26.5	47	17.4
Male	367	73.5	223	82.6
**Histologic grade**				
G1–2	359	71.9		
G3–4	121	24.2		
Gx	16	3.2		
NA	3	0.6		
**Stage**				
I–II	95	19.0	55	20.4
III–IV	337	67.5	215	79.6
NA	67	13.4		
**T classification**				
T1–2	177	35.2	115	42.6
T3–4	267	53.5	155	57.4
Tx	33	6.6		
NA	22	4.4		
**N classification**				
N0	170	34.1	94	34.8
N+	236	47.3	176	65.2
Nx	69	13.8		
NA	24	4.8		
**M classification**				
M0	185	37.1	263	97.4
M1	1	0.2	7	2.6
Mx	61	12.2		
NA	252	50.5		
**Vital status**				
Deceased	218	43.7	94	34.8
Living	281	56.3	176	65.2

### Identification of Differentially Expressed Genes (DEGs)

The differentially expressed RBPs (DERBPs) between SCCHN and normal samples were evaluated utilizing the Wilcoxon test by *limma* R package. We determined cutoff values according to the false discovery rate (FDR) (Nakamura et al., 2010) and defined RBPs with FDR < 0.05 and | logFC| > 1 as significant DERBPs. Subsequently, we constructed a heat map by the *pheatmap* R package and a volcano plot to show the DERBPs. The distributions of DERBPs on chromosomes were displayed using the *OmicCircos* R package (Hu et al., 2014).

### GO and KEGG Pathway Analyses

To analyze the function of DERBPs, Gene Ontology (GO) and Kyoto Encyclopedia of Genes and Genomes (KEGG) pathway enrichment analyses were performed by the *enrichplot* R package (Yu et al., 2012). GO terms included biological process (BP), cellular component (CC), and molecular function (MF). For the analysis results, both *P*-value and FDR < 0.05 were defined as statistical significance.

### Protein–Protein Interaction (PPI) Network Construction

We constructed the PPI network of DERBPs to investigate protein interactions using STRING (version 11.0)^4^ (Szklarczyk et al., 2019) according to a combined score >0.4. Then, the PPI network was visualized by Cytoscape software (version 3.7.1) (Smoot et al., 2011). Furthermore, the Molecular Complex Detection (MCODE, version 1.6.1) (Bader and Hogue, 2003) plug-in in Cytoscape was utilized to screen the key modules based on Degree Cutoff = 2, Node Score Cutoff = 0.2, K-Core = 2.

### Construction of a Prognostic Risk Model

To identify overall survival (OS)-associated DERBPs, we performed univariate Cox regression analysis. We chose the candidate prognostic genes according to *P*-value < 0.05. Subsequently, the multigene prognostic risk model was constructed by Lasso regression analysis in the TCGA training set. We calculated the risk score of each patient using the regression coefficients of each candidate gene according to the following computational formula:

$$R\,i\,s\,k\,s\,c\,o\,r\,e=\sum_{k=1}^{n}C\,o\,e\,f\,\left(g\,e\,n\,e_{k}\right)*E\,x\,p_{k}$$

Here, *n* is the number of the candidate genes of the prognostic risk model, *gene*_*k*_ is the *_*k*_*th candidate gene, *Coef* is the estimated regression coefficient of the candidate genes from the Lasso regression analysis, and *Exp*_*k*_ is the mRNA expression level of the *_k_*th candidate gene. Then, we clustered the SCCHN patients into high-risk and low-risk groups with the median the risk score of the TCGA training set. The association between the candidate genes and risk scores were shown using the hierarchical cluster heat map.

### Gene Set Enrichment Analysis

Gene set enrichment analysis (GSEA) is an analytical method used to estimate significant differences between two biological conditions to determine specific functional gene sets (Subramanian et al., 2005). In our research, GSEA was performed utilizing GSEA (version 4.0.3)^5^ with the Molecular Signatures Database (MSigDB) (Liberzon et al., 2011). C2 curated gene sets, and a list of significantly different gene sets between the high-risk and low-risk groups was generated. Gene sets, performed 1,000 times for each analysis, with *p* < 0.05 and FDR < 0.25 were defined as significantly enriched.

### Evaluation of Immune Scores and Immune Cell Infiltration

The ESTIMATE (Estimation of Stromal and Immune cells in Malignant Tumor tissues using Expression data) algorithm is a method used to calculate the immune and stromal scores of tumor samples. The immune and stromal scores of SCCHN samples TCGA data set was calculated by the *estimate* R package (Yoshihara et al., 2013).

In addition, we assessed the composition fraction of tumor-infiltrating immune cells of each SCCHN sample by CIBERSORT^6^. CIBERSORT is an algorithm used to characterize the cell composition of complex tissues according to gene expression profiles (Newman et al., 2015).

### Statistical Analysis

All statistical analyses were performed utilizing R project (version 3.6.3). Wilcoxon rank-sum test was a non-parametric statistical hypothesis test mainly used for comparisons between two groups and Kruskal–Wallis test was suitable for two or more categories. Survival analysis was estimated using the Kaplan–Meier curve with the log-rank test. The diagnostic values of the risk score and other clinical factors were evaluated utilizing ROC curve analysis. The correlation between the variables was identified by Spearman’s rank correlation test. *P* < 0.05 was identified as statistically significant.

## Results

### Analysis of Differentially Expressed RBPs in SCCHN Samples

**FIGURE 1:**
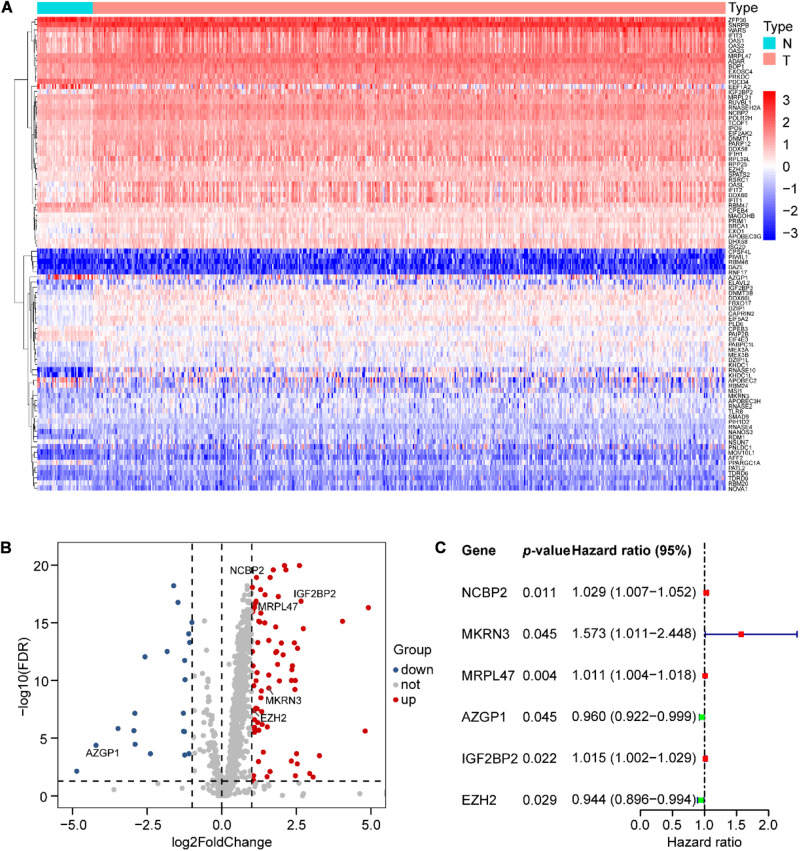
Differential expression of RNA-binding proteins (RBPs) and six RBPs of prognostic risk models in SCCHN samples. **(A)** Ninety-two differential expression of RBPs (DERBPs) displayed by the heat map. **(B)** Seventy-four upregulated and eighteen downregulated DERBPs shown by the volcano plot (FDR < 0.05 and | logFC| > 1). **(C)** Characteristics of six risk DEARGs in the prognostic risk model exhibited by the forest plot.

We analyzed the expression profiles of 1,542 human RBPs (Supplementary Table 3), distributed on all chromosomes, including sex chromosomes X and Y (Supplementary Figure 1), in 498 SCCHN with 44 normal tissues in the TCGA data set. Then, we identified 92 differentially expressed RBPs (DERBPs), including 74 upregulated and 18 downregulated DERBPs (FDR < 0.05 and |logFC| > 1). The DERBPs are listed in Supplementary Table 4 and are visualized by the heat map (Figure 1A) and the volcano plot (Figure 1B).

### Function Analysis of DERBPs in the TCGA Data Set

**FIGURE 2:**
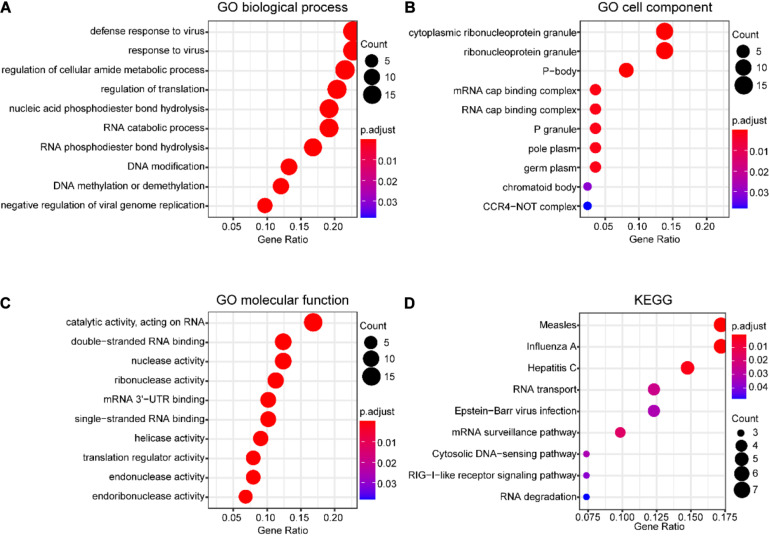
Functional enrichment analysis of DERBPs in SCCHN of the TCGA data set. **(A–C)** The top 10 enriched GO terms of biological process **(A)**, cell component **(B)**, and molecular function **(C)** for DEUPSGs shown using a scatter diagram. **(D)** The enriched KEGG pathways also demonstrated using a scatter diagram. GO, gene ontology; KEGG, Kyoto Encyclopedia of Genes and Genomes.

The potential function of DERBPs in the TCGA data set was analyzed utilizing GO and KEGG pathway enrichment analyses. The top 10 enriched GO terms of BP, CC, and MF for DERBPs were displayed using the scatter plot (Figures 2A–C). The most significant enriched terms of BP, CC, and MF were associated with defense response to virus, cytoplasmic ribonucleoprotein granule, and catalytic activity, acting on RNA, respectively. The enriched pathways of KEGG pathway analysis were also demonstrated with the scatter plot (Figure 2D). The results showed that the DERBPs might be related to measles, influenza A, hepatitis C, RNA transport, Epstein–Barr virus infection, mRNA surveillance pathway, cytosolic DNA-sensing pathway, RIG-I-like receptor signaling pathway, and RNA degradation. The functional analyses revealed that the DERBPs are mainly related to RNA metabolism.

### PPI Network Construction

**FIGURE 3:**
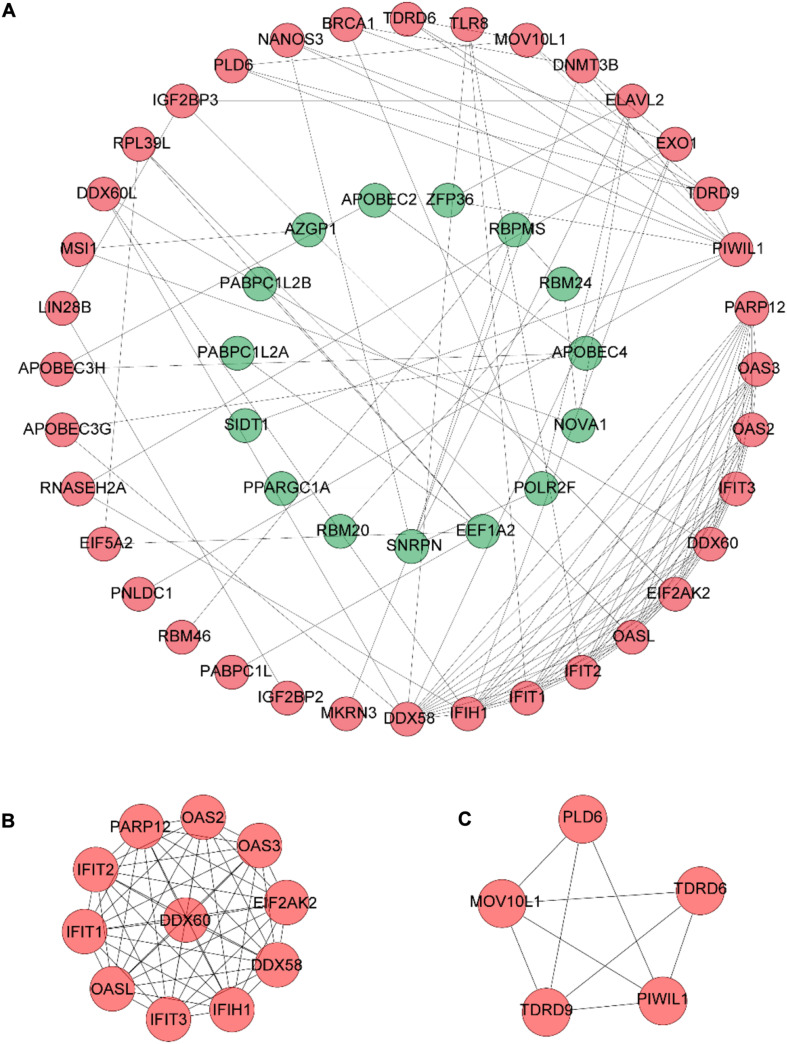
PPI network construction. **(A)** PPI network constructed with the DERBPs of the TCGA data set. **(B,C)** The top two modules (>5 nodes), module 1 **(B)** and module 2 **(C)**, in the PPI network.

The PPI network of DERBPs was constructed using STRING according to combined scores > 0.4, and then the results were visualized by Cytoscape software (Figure 3A), in order to better understand the potential interactions among DERBPs. In addition, the key modules of the PPI network were screened utilizing MCODE and two modules were selected (Figures 3B,C).

### Identification of a Prognostic Risk Model in the TCGA Training Set

To identify prognostic DERBPs of SCCHN patients, the expression profiles of the 92 DERBPs in the TCGA training set were analyzed using univariate Cox regression analysis. Moreover, six prognosis-associated DERBPs, including NCBP2, MKRN3, MRPL47, AZGP1, IGF2BP2, and EZH2, of the TCGA training set are exhibited by forest plot (Figure 1C). Then, a prognostic risk model of six prognosis-associated DERBPs was constructed utilizing LASSO regression analysis (Supplementary Figure 2). The information and the coefficient values of the six genes are shown in Table 2. The prognostic risk score of each SCCHN patient was calculated according to the following formula:

**TABLE 2:** The list of the six RBP genes of the prognostic risk model in SCCHN.

ENSG ID	Symbol	Location	Expression status	Coefficient
ENSG00000114503	NCBP2	Chromosome 3	Upregulated	0.0215
ENSG00000179455	MKRN3	Chromosome 15	Upregulated	0.3196
ENSG00000136522	MRPL47	Chromosome 3	Upregulated	0.0086
ENSG00000160862	AZGP1	Chromosome 7	Downregulated	–0.0301
ENSG00000073792	IGF2BP2	Chromosome 3	Upregulated	0.0013
ENSG00000106462	EZH2	Chromosome 7	Upregulated	–0.0842

*Risk score* = NCBP2* *0.0215* + *MKRN3* ^∗^ *0.3196* + *MRPL47* ^∗^ *0.0086* + *AZGP1* ^∗^ *(−0.0301)* + IGF2BP2* *0.0013* + *EZH2* ^∗^ *(−0.0842)*

**FIGURE 4:**
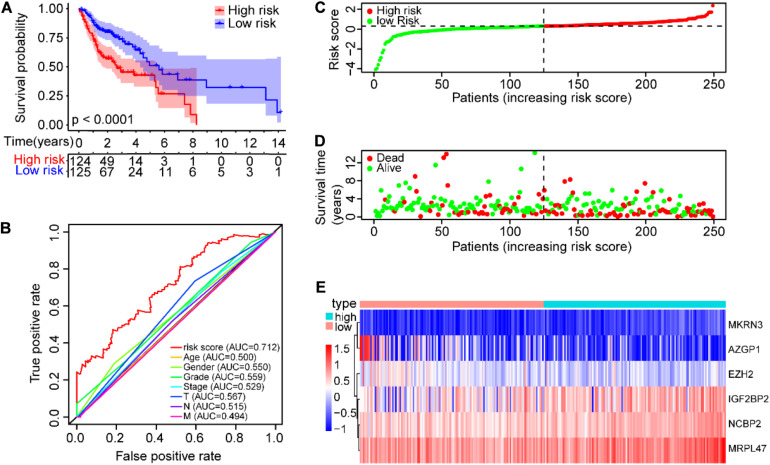
Construction of the prognostic risk model of SCCHN patients in the TCGA training set. **(A)** Kaplan–Meier survival curve with OS of SCCHN patients in the high-risk and low-risk groups in the TCGA training set. **(B)** ROC curve demonstrating AUC value of the risk score and other clinical parameters of SCCHN patients. **(C)** The risk plot distribution of SCCHN patients with high-risk and low-risk scores. **(D)** The survival status of SCCHN patients. **(E)** The expression patterns of the six genes of the risk model in TCGA training set.

The SCCHN patients in the TCGA training set were divided into low-risk and high-risk groups according to the median cutoff value of risk scores (0.2962). Survival analysis demonstrated that the overall survival (OS) of the high-risk group was significantly worse than that of the low-risk group (*P* < 0.0001, Figure 4A). The receiver operating characteristic (ROC) curve analysis showed that the area under the ROC (AUC) value was 0.712, higher than other clinical factors (Figure 4B). The risk scores and survival status of SCCHN patients in the training set were ranked with dot plots (Figures 4C,D). The expression patterns of six genes in the high-risk and low-risk groups of the training set are demonstrated using the heat map (Figure 4E), which indicated that high expressions of NCBP2, MKRN3, MRPL47, and IGF2BP2 serve as risk factors associated with the high-risk score, while high expressions of AZGP1 and EZH2 act as protective factors associated with the low-risk score.

### Verification of the Prognostic Risk Model in the TCGA Data Sets

**FIGURE 5:**
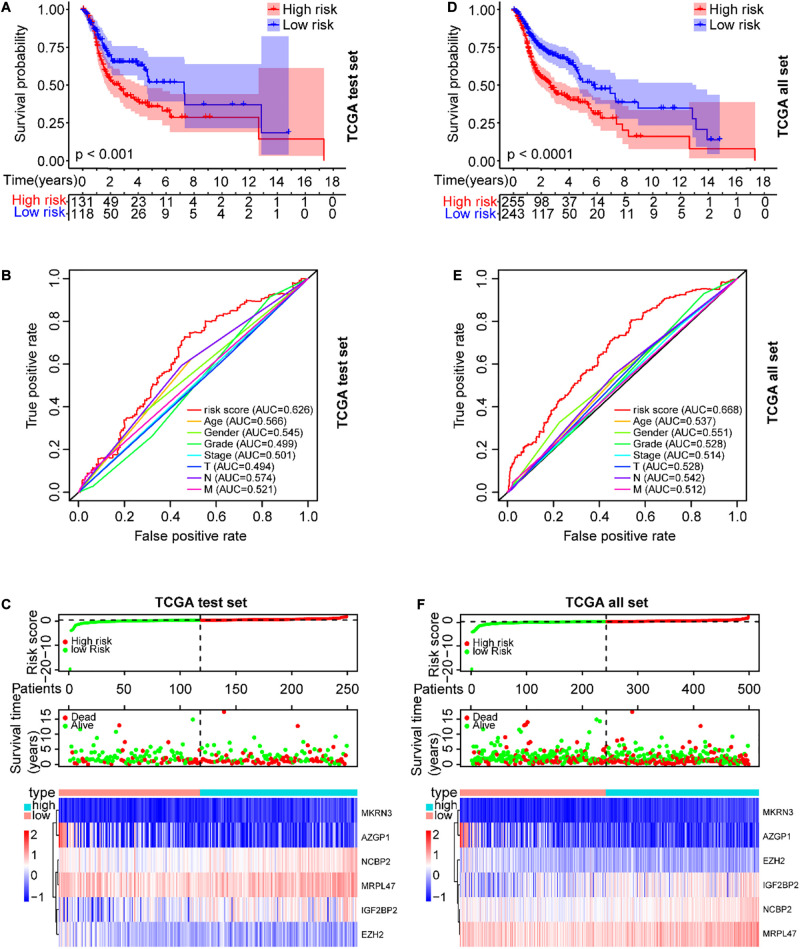
Verification of the prognostic risk model in the TCGA data sets. **(A)** The OS of SCCHN patients in the risk subgroups showed by the Kaplan–Meier curve in the TCGA test set and the TCGA all set, respectively. **(B)** The AUC value of risk score and other clinical parameters in the TCGA test set using the ROC curve analysis. **(C)** The risk plot distribution, survival status, and expression patterns of risk genes of SCCHN patients in the TCGA test set. **(D–F)** A similar analysis performed in the TCGA data set corresponding to the TCGA test set.

To validate the prognostic risk model, independent validation data sets were used to test. According to the risk model from the training data set, all SCCHN patients in the TCGA test data set were also segregated into high-risk and low-risk groups. Kaplan–Meier curve analysis showed the survival of the high-risk group was worse than that in the low-risk group (*P* < 0.001, Figure 5A). The AUC value of the risk score in the TCGA test set was 0.626 using the ROC curves analysis, higher than other clinical parameters (Figure 5B). The association between the expression patterns of the six risk genes and the risk score was consistent with the training set (Figure 5C). A similar analysis also was performed in the TCGA data set; the results were also consistent with the training set (Figures 5D–F).

### Verification of the Prognostic Risk Model in the GEO Data Set

**FIGURE 6:**
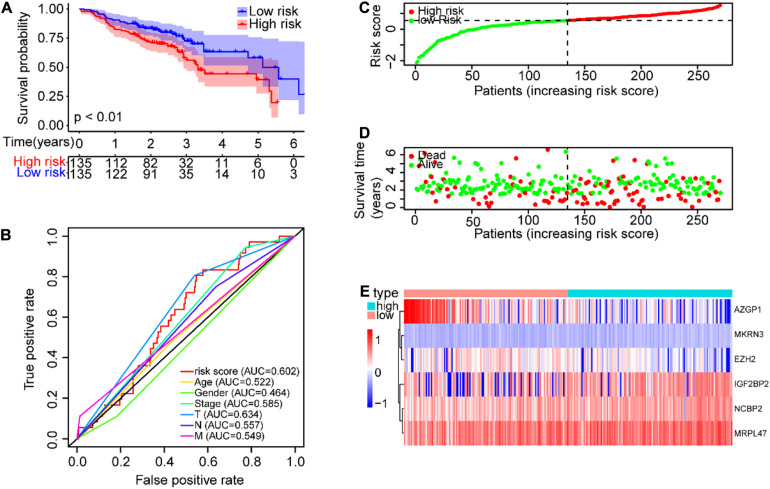
Validation of the risk model in GEO (GSE65858) data set. **(A)** Kaplan–Meier survival curve with OS of SCCHN patients in the risk subgroups of the GSE65858 (GEO) data set. **(B)** ROC curve showing the AUC value of the risk score and other clinical factors of SCCHN patients in the GSE65858 data set. **(C)** The risk score distribution of SCCHN patients in the high-risk and low-risk groups. **(D)** Scatter plot showing the survival status of SCCHN patients. **(E)** The expression patterns of risk genes of SCCHN samples in the GSE65858 data set.

Further, the GEO data set (GSE65858) with 270 SCCHN patients was used as an external independent data set to validate the risk model. The patients in the GEO test set were also classified into high-risk and low-risk groups, and the prognoses of the high-risk group were also significantly worse than those of the low-risk group (*P* < 0.0001, Figure 6A). The AUC value of the risk score was 0.602, also higher than other clinical parameters, except for T classification (Figure 6B). The risk scores and survival status of SCCHN patients were also shown with dot plots (Figures 6C,D). The association between the expression profiles of the six genes and the risk score in the GEO data set was also in line with the training set (Figure 6E).

### Association Between the Risk Score and the Clinical Parameters of SCCHN Patients

**FIGURE 7:**
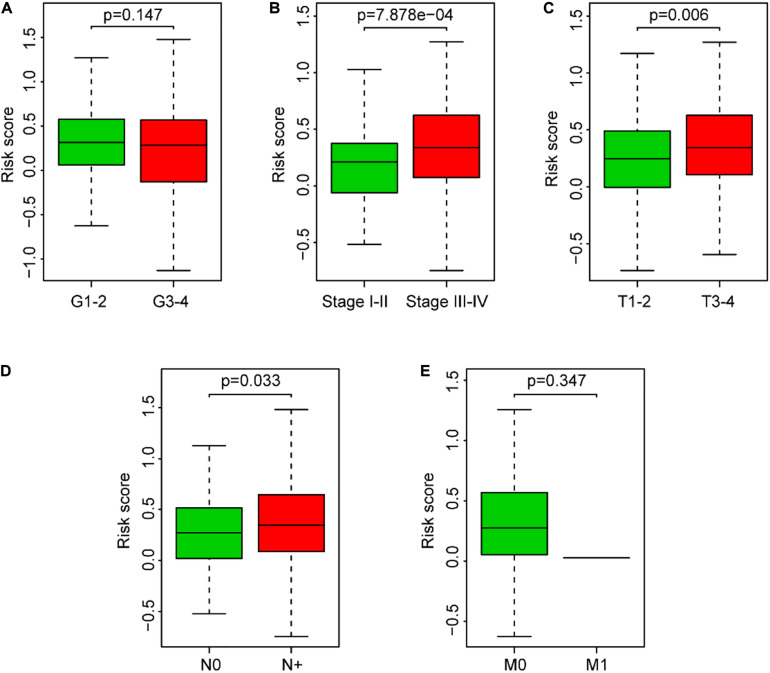
Association between the risk score and clinical characteristics of SCCHN patients. **(A)** Subgroup analysis of pathology grade (Grades 1–2 vs. Grades 3–4). **(B)** Subgroup analysis of clinical stage (Stages I–II vs. Stages III–IV). **(C)** Subgroup analysis of T classification (T1–2 vs. T3–4). **(D)** Subgroup analysis of N classification (N0 vs. N+). **(E)** Subgroup analysis of M classification (M0 vs. M1).

The clinical parameter subgroup analysis of the risk score was shown (Figures 7A–E and Table 3), and the results revealed that the risk score of SCCHN patients with stages III–IV, T3–4, and N + were higher than that with stages I–II, T1–2, and N0, respectively (*P* < 0.0001, *P* < 0.01, and *P* < 0.05, respectively). However, the risk scores between the subgroups of grade and M classification were not statistically significant (*P* = 0.147 and *P* = 0.347, respectively). In addition, we analyzed the association between the risk score and other clinical parameters using logistic regression in the TCGA data set (Table 4). The level of risk score was significantly associated with clinical stage (*P* < 0.01), T classification (*P* < 0.05), and N classification (*P* < 0.05). However, it was not correlated with other clinical parameters, including age (*P* = 0.817), gender (*P* = 0.234), histological grade (*P* = 0.344), and M stage (*P* = 0.347). These results suggested that the risk score was closely associated with the progression of SCCHN.

**TABLE 3:** Association analysis between the clinical factors and the risk score in SCCHN patients of TCGA data set using logistic regression.

Clinical characteristics	Total (N)	Odds ratio in the risk score	*p*-value
Age (≥60 vs. <60)	498	1.043 (0.731–1.487)	0.817
Gender	498	1.275 (0.856–1.904)	0.234
Grade (G1–2 vs. G3–4)	478	0.819 (0.541–1.238)	0.344
Stage (I–II vs. III–IV)	430	2.185 (1.367–3.542)	0.001
T classification (T1–2 vs. 3–4)	442	1.633 (1.113–2.404)	0.012
N classification (N0 vs. N+)	404	1.515 (1.019–2.258)	0.041

**TABLE 4:** Gene sets enriched in the high-risk and low-risk groups.

MSigDB collection	Name	NES	ES	NOM *p*-val	FDR *q*-val
c2.cp.kegg.v7.1.symbols.gmt	KEGG_PROTEASOME	1.950	0.724	0.004	0.079
	KEGG_PROTEIN_EXPORT	1.733	0.632	0.019	0.214
	KEGG_ARACHIDONIC_ACID_METABOLISM	–2.141	–0.593	0.000	0.007
	KEGG_T_CELL_RECEPTOR_SIGNALING_PATHWAY	–2.083	–0.619	0.002	0.006
	KEGG_FC_EPSILON_RI_SIGNALING_PATHWAY	–1.968	–0.544	0.002	0.029
	KEGG_B_CELL_RECEPTOR_SIGNALING_PATHWAY	–1.924	–0.579	0.006	0.042
	KEGG_FC_GAMMA_R_MEDIATED_PHAGOCYTOSIS	–1.901	–0.521	0.004	0.046
	KEGG_LINOLEIC_ACID_METABOLISM	–1.880	–0.620	0.006	0.053
	KEGG_CHEMOKINE_SIGNALING_PATHWAY	–1.867	–0.517	0.010	0.052
	KEGG_NATURAL_KILLER_CELL_MEDIATED_CYTOTOXICITY	–1.830	–0.526	0.018	0.058
	KEGG_CYTOKINE_CYTOKINE_RECEPTOR_INTERACTION	–1.691	–0.462	0.024	0.099
	KEGG_FATTY_ACID_METABOLISM	–1.605	–0.523	0.035	0.129

### GSEA Analysis of the Risk Score-Associated Signaling Pathway

**FIGURE 8:**
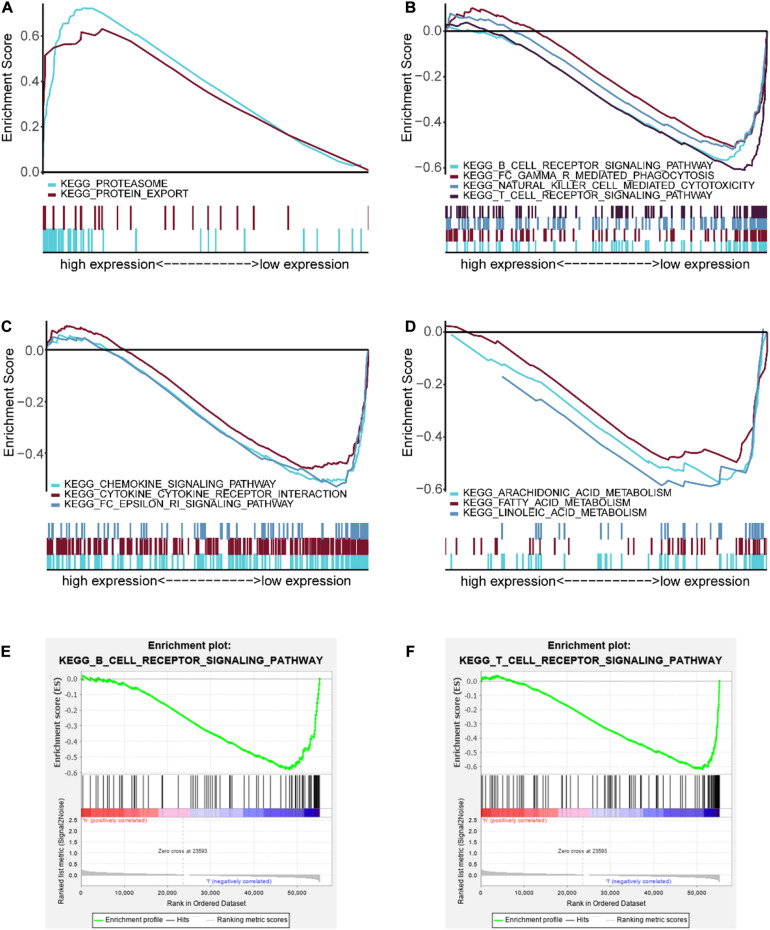
GSEA-enriched pathways of the high-risk and low-risk groups. **(A)** Multiple GSEA-enriched pathways of the high-risk group: proteasome and protein export. **(B)** Multiple GSEA for immune-related pathways of the low-risk group: B cell receptor signaling pathway, Fc gamma R-mediated phagocytosis, natural killer cell mediated cytotoxicity, and T cell receptor signaling pathway. **(C)** Multiple GSEA for inflammatory response-related pathways of the low-risk group: chemokine signaling pathway, cytokine–cytokine receptor interaction, and Fc epsilon RI signaling pathway. **(D)** Multiple GSEA for fatty acid metabolism-related pathways of the low-risk group: arachidonic acid metabolism, fatty acid metabolism, and linoleic acid metabolism. **(E)** Single GSEA showing the B cell receptor signaling pathway of the low-risk group. **(F)** Single GSEA showing the T cell receptor signaling pathway of the low-risk group.

GSEA analysis was performed to unravel significantly enriched pathways of the high-risk and low-risk groups in the TCGA data set. The top 10 enriched pathways of the high-risk group and thirty enriched pathways of the low-risk group were demonstrated (Supplementary Table 5). Enriched pathways with significant differences (FDR < 0.25, NOM *p* < 0.05) were selected (Table 4). The results demonstrated that protein degradation and export related pathways were significantly enriched in the high-risk group (Figure 8A); however, immune, inflammatory response and fatty acid metabolism were significantly enriched in the low-risk group (Figures 8B–D). Intriguingly, the B cell receptor signaling pathway and T cell receptor signaling pathway were enriched in the low-risk group (Figures 8E,F), which indicated that the high-risk score may be associated with immunosuppression. Other individual GSEA plots are shown in Supplementary Figure 3.

### Association Between the Risk Score and Tumor Immunity

**FIGURE 9:**
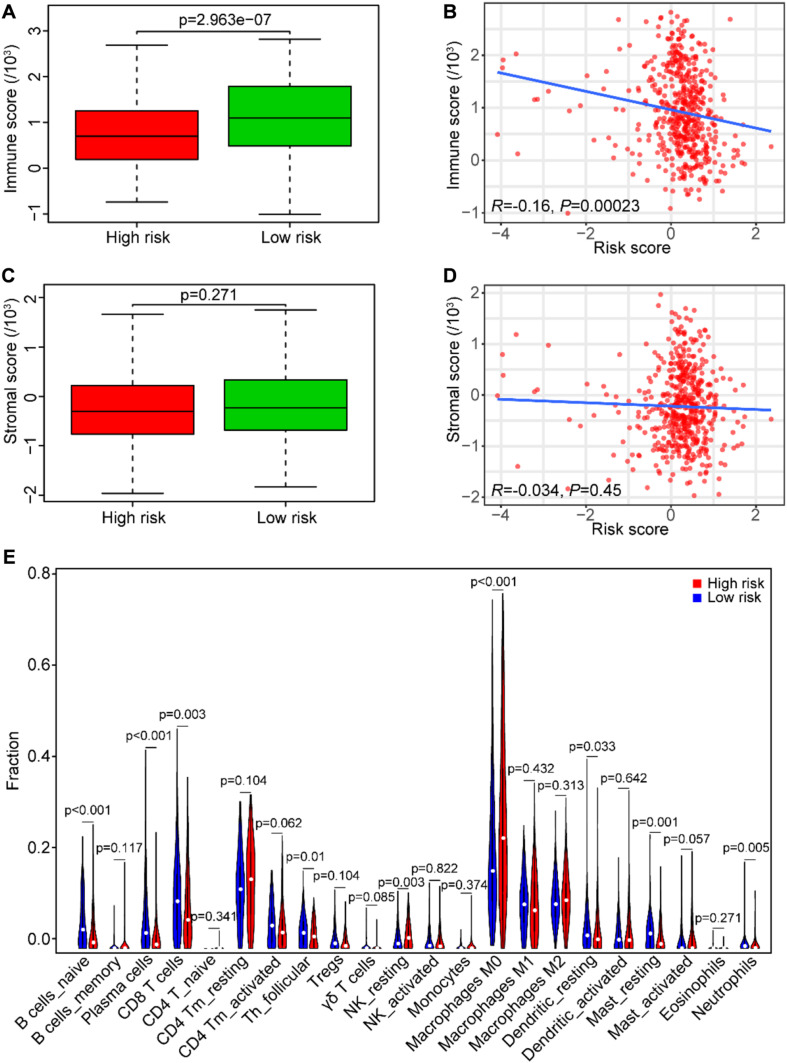
Association between the risk score and tumor immunity in the TCGA data set. **(A)** The immune score distribution in risk subgroups of SCCHN patients. **(B)** Correlation of the risk score with the immune score in SCCHN samples. **(C)** The stromal score distribution in risk subgroups of SCCHN patients. **(D)** Correlation of the risk score with the stromal score in SCCHN samples. **(E)** Comparison fractions of immune cells between the high-risk and low-risk groups.

For the hint from the GSEA results that the high-risk score may be associated with tumor immunosuppression, we performed the ESTIMATE to identify the immune/stromal score of the TCGA data set. Our results showed that tumor samples in the low-risk group had higher immune scores than those in the high-risk group (*P* < 0.0001, Figure 9A). In addition, the risk score was significantly and negatively correlated with the immune score in SCCHN samples by Spearman’s rank test (*R* = *–*0.16, *P* < 0.001, Figure 9B), and there was no significant correlation between the risk score and the stromal score in SCCHN samples (Figures 9C,D).

Moreover, we identified the composition of infiltrating immune cells of SCCHN samples in the TCGA data set using the CIBERSORT to analyze immune cells between the risk subgroups (Figure 9E). Consistent with the GSEA results, the result revealed that SCCHN samples in the high-risk group contained a lower fraction of naïve B cells (*P* < 0.001), CD8 T cells (*P* < 0.01), and follicular helper T (*P* < 0.05) compared with those in the low-risk group. These results suggested that the high-risk score was associated with immunosuppression.

### Correlation of the Genes of the Risk Model With the Three Immune Cell Types

**FIGURE 10:**
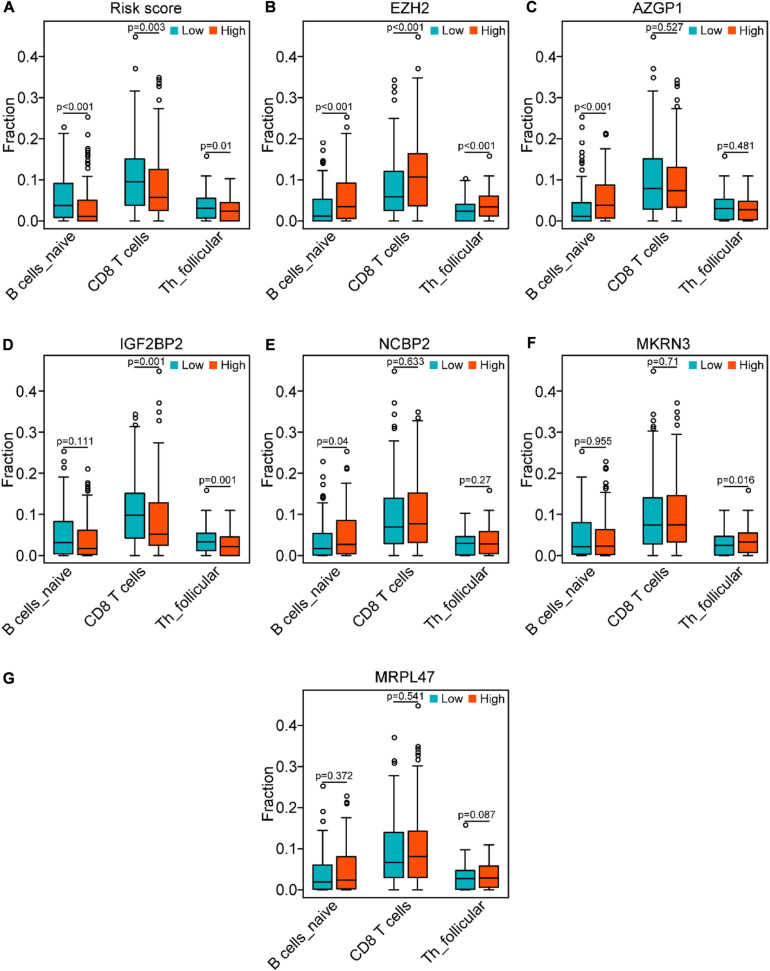
Correlation of the genes of the risk model with the three immune cells in the TCGA data set. **(A)** Comparison of the three immune cells (naïve B cells, CD8 T cells, and follicular helper T cells) between the high-risk and low-risk groups. **(B–G)** Distribution of the three immune cells in the subgroups with high or low expression of EZH2, AZGP1, IGF2BP2, NCBP2, MKRN3, and MRPL47, respectively.

In line with the association between the risk score and the above three immune cell types (Figure 10A), we investigated the association between three types of immune cells with the expression levels of six genes in the risk model (Figures 10B–G). Consistently with the expression patterns of the risk genes, the reduction of naïve B cells was associated with the low expression of EZH2 (*P* < 0.001) and AZGP1 (*P* < 0.001). In addition, the decrease of CD8 T cells was related to the low expression of EZH2 (*P* < 0.001) and the high expression of IGF2BP2 (*P* < 0.01). Moreover, the asthenia of follicular helper T cells was associated with the low expression of EZH2 (*P* < 0.001) and the high expression of IGF2BP2 (*P* < 0.01). Hence, the risk genes EZH2, AZGP1, and IGF2BP2 play key roles in immunosuppression of SCCHN.

## Discussion

Evasion of immune destruction of cancer cells is one key hallmark of cancer (Hanahan and Weinberg, 2011), and RBPs can regulate the function and activity of immune cells, which eventually may be linked to immune surveillance evasion of cancer cells through managing the RNA metabolism at the post-transcription level (Kafasla et al., 2014; Pereira et al., 2017). In addition, accumulating studies have reported that dysregulated expression of RBP-facilitated cell proliferation, invasion, and metastasis and pluripotency and stemness in multiple cancers (Yu et al., 2007; Dong et al., 2019; Soni et al., 2019; Velasco et al., 2019; Elcheva et al., 2020; Pascual et al., 2020). However, few studies have analyzed the expression patterns and roles of RBPs in SCCHN. In our study, for the first time, we comprehensively analyzed the expression patterns and potential functions of RBPs in SCCHN.

Here, we initially comprehensively analyzed the associations between the prognosis of SCCHN and 92 differentially expressed RBPs (DERBPs, Figures 1A,B). Subsequently, we constructed a prognosis risk model in the training set with six RBPs, including NCBP2, MKRN3, MRPL47, AZGP1, IGF2BP2, and EZH2 (Figure 1C), which showed a robust performance for predicting prognosis compared with clinical parameters in training and multiple validation sets (Figures 4–6).

In this prognostic risk model, AZGP1 and EZH2 served as protective factors, while NCBP2, MKRN3, MRPL47, and IGF2BP2 acted as risk factors. Low AZGP1 expression was significantly associated with increased risk of biochemical relapse in margin-positive localized prostate cancer (Yip et al., 2011; Bruce et al., 2016). AZGP1 as a cancer suppressor inhibited cell proliferation, migration, and invasion via TGF-β and PTEN/Akt signaling pathways in pancreatic cancer and hepatocellular carcinoma (Kong et al., 2010; Tian et al., 2017). These studies consistently with our results suggested AZGP1 as an anticancer gene. Previous studies have shown that EZH2 silencing reduced cancer cell growth, migration and invasion in SCCHN (Liu et al., 2013; Chang et al., 2016), but which lack transgenic animal experiments and does not involve the influence of EZH2 on tumor microenvironment regulation of tumor genesis and development. IGF2BP2 overexpression was observed in pancreatic cancer, colorectal cancer, and SCCHN, which promoted cancer cell proliferation by activating the PI3K/Akt signaling pathway (Ye et al., 2016; Wang et al., 2019; Xu et al., 2019; Deng et al., 2020). These studies consistently with our results suggested that IGF2BP2 served as an oncogene. However, the roles of NCBP2, MKRN3, and MRPL47 in cancers are still unclear. Our study was first to suggest that they can be acted as risk factors in a prognostic risk model of SCCHN.

Interestingly, our GSEA results unveiled that the B cell receptor (BCR) signaling pathway and the T cell receptor (TCR) signaling pathway enriched in the low-risk group (Figures 8E,F), which indicated that they may be asthenic in the high-risk group. BCR and TCR signalings have been shown pivotal for B cell and T cell proliferation and development for adaptive immunity, and their abnormalities could lead to immunodeficiency (Burger and Wiestner, 2018; Tan et al., 2019; Berry et al., 2020; Takeuchi et al., 2020). Therefore, we identified the correlation between the risk score and the immune score and analyzed the composition of immune cells between the risk subgroups in SCCHN samples of the TCGA data set. As we confirmed, there was a negative correlation between the high-risk score and tumor immune score, and the high-risk group contained lower fractions of naïve B cells, CD8 T cells, and follicular helper T cells compared with the low-risk group (Figure 9). Hence, the results revealed that the high-risk score may be an essential factor for B and T cell growth and differentiation leading to tumor immunosuppression, and the low expression of EZH2 and AZGP1 and high expression of IGF2BP2 were the main factors of tumor immunosuppression in the risk model (Figure 10). During the humoral immune response, EZH2 expression was remarkably elevated in the B cells of the germinal center (GC) (Caganova et al., 2013; Herviou et al., 2019), which directly inhibited cell cycle inhibitors of B cells, including CDKN1A (Béguelin et al., 2013, 2017). Similar to B cell development, EZH2 promoted generation and differentiation of mature T lymphocytes via preventing p53 stabilization to suppress CNKN2 (Jacobsen et al., 2017). In addition, EZH2 depletion in CD8^+^ T cells restrained the amplification of antigen-specific effector cells after pathogenic microorganisms infection (Gray et al., 2017; Chen et al., 2018). Albeit the roles of AZGP1 and IGF2BP2 in the immune response have not been investigated, our study was first to suggest that AZGP1 downregulation and IGF2BP2 upregulation may act as suppressors in tumor immune response in SCCHN.

Although we identified a prognostic risk model with six RBPs and revealed that the high-risk score was significantly associated with cancer immunosuppression, the results of our study performed with bioinformatics analysis were not robust enough needing to be confirmed utilizing experimental approaches. Thus, multicenter studies with larger sample sizes are required.

## Conclusion

In summary, in our study, we developed a robust prognostic risk model with six differentially expressed RBPs. The results showed that the risk score has great potential as a prognostic and immunosuppression state biomarker in SCCHN patients. Therefore, the risk model may act as a prognostic signature and offer highlights for individualized immunotherapy of SCCHN patients.

## Data Availability Statement

Publicly available datasets were analyzed in this study. This data can be found here: The Cancer Genome Atlas (TCGA) (https://portal.gdc.cancer.gov/) and gene expression omnibus (GEO) database GSE65858 (https://www.ncbi.nlm.nih.gov/geo/).

## Author Contributions

ZL contributed to this project design and final approval of the manuscript. GH, JYa, QJ, LL, HP, YW, SL, YT, and JYu performed the data analysis, interpretation, visualization, and drafting. All authors contributed to the article and approved the submitted version.

## Conflict of Interest

The authors declare that the research was conducted in the absence of any commercial or financial relationships that could be construed as a potential conflict of interest.

## Footnotes

1 https://portal.gdc.cancer.gov/

2 https://www.ncbi.nlm.nih.gov/geo/

3 https://www.r-project.org/

4 https://string-db.org/

5 https://www.gsea-msigdb.org/gsea/downloads.jsp

6 http://cibersort.stanford.edu/

## Supplementary Material

Supplementary Figure 1 The distribution of the DERBPs on chromosomes. A total of 1,542 human RBPs distributing on all chromosomes, including sex chromosome X and Y.

Supplementary Figure 2 Construction of a prognostic risk model using LASSO regression analysis. **(A)** Prognostic-related DERBPs identified by LASSO algorithms in the TCGA training set. **(B)** The prognostic risk model constructed with LASSO coefficient values in the TCGA training set.

Supplementary Figure 3 Other single GSEA figures of the high-risk and low-risk groups. Single GSEA plots showing enriched pathways in the high-risk and low-risk groups displayed in Table 3, except the B cell receptor signaling pathway and T cell receptor signaling pathway.

Supplementary Table 1 The patient information of TCGA training set.

Supplementary Table 2 The patient information of TCGA test set.

Supplementary Table 3 The list of 1,542 RBP genes.

Supplementary Table 4 Ninety-two differentially expressed RBP genes.

Supplementary Table 5 The top 10 and 30 enriched pathways of the high and low risk group, respectively.
