# Supplementary figures and images for: Integrated Analysis of RNA-Binding Proteins Associated With the Prognosis and Immunosuppression in Squamous Cell Carcinoma of Head and Neck
Source: Front Genet. 2021 Jan 11;11:571403. doi: 10.3389/fgene.2020.571403 (PMC7831273; doi:10.3389/fgene.2020.571403)

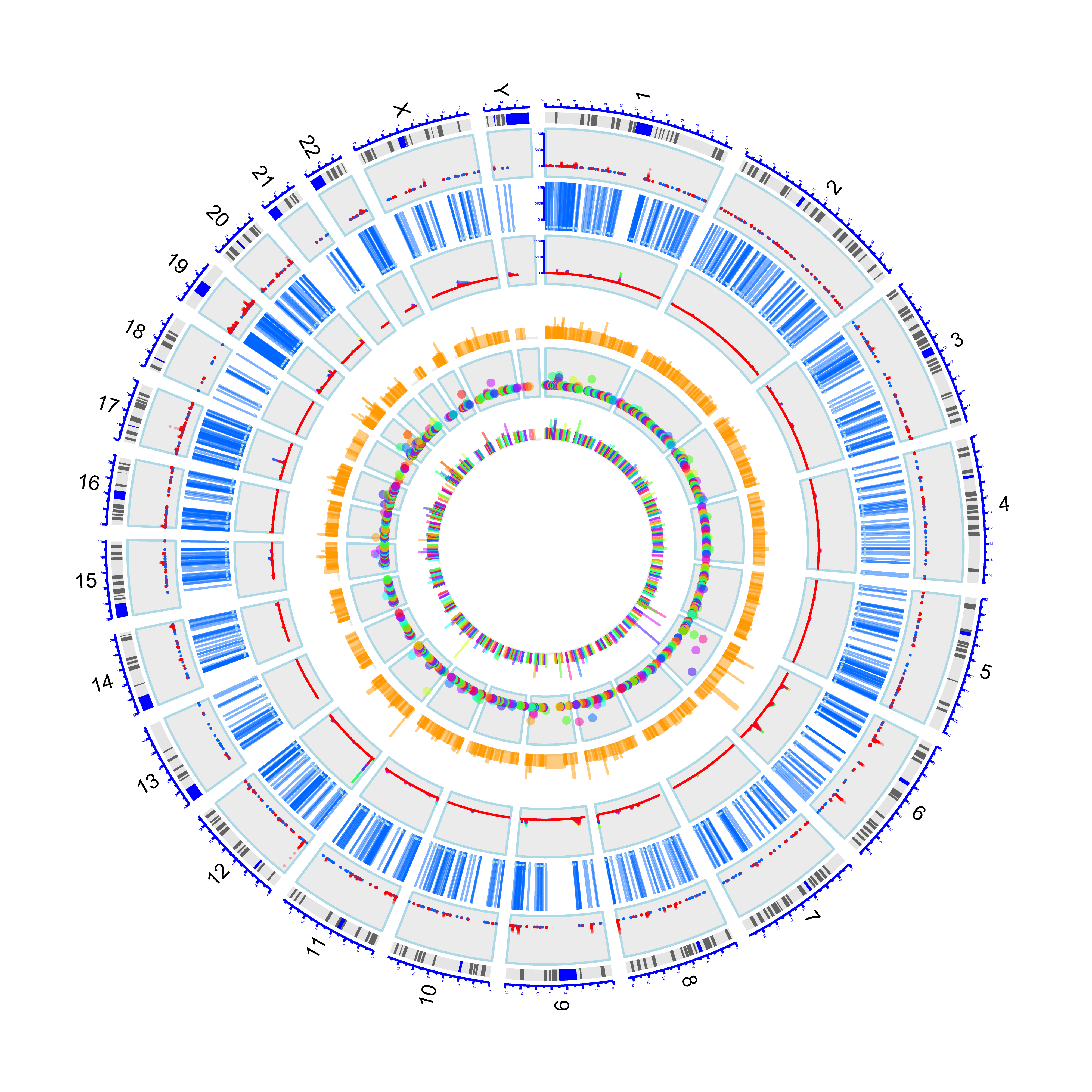

Supplement: Supplementary Figure 1 — The distribution of the DERBPs on chromosomes. A total of 1,542 human RBPs distributing on all chromosomes, including sex chromosome X and Y. [file Image_1.TIF]

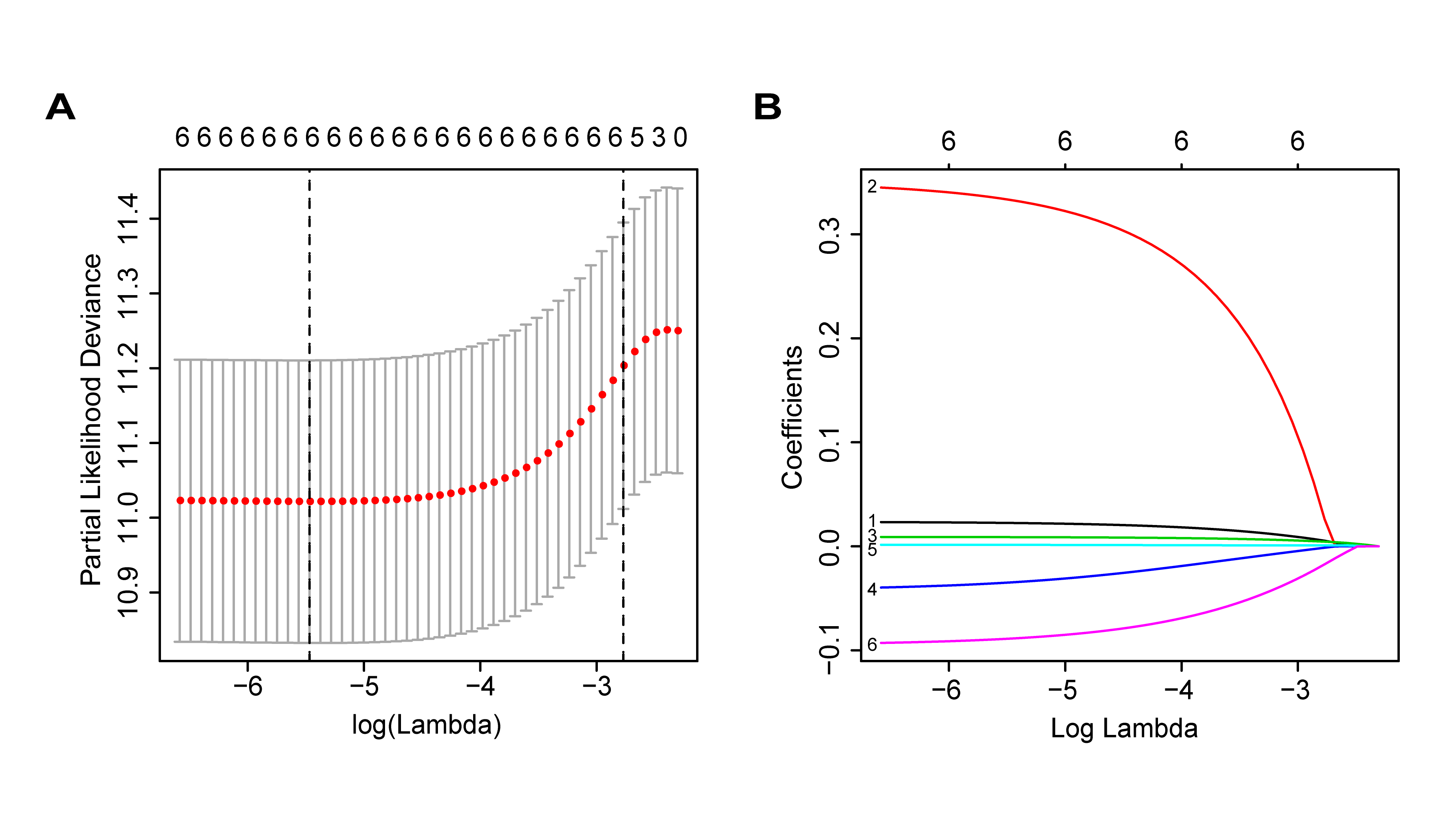

Supplement: Supplementary Figure 2 — Construction of a prognostic risk model using LASSO regression analysis. (A) Prognostic-related DERBPs identified by LASSO algorithms in the TCGA training set. (B) The prognostic risk model constructed with LASSO coefficient values in the TCGA training set. [file Image_2.TIF]

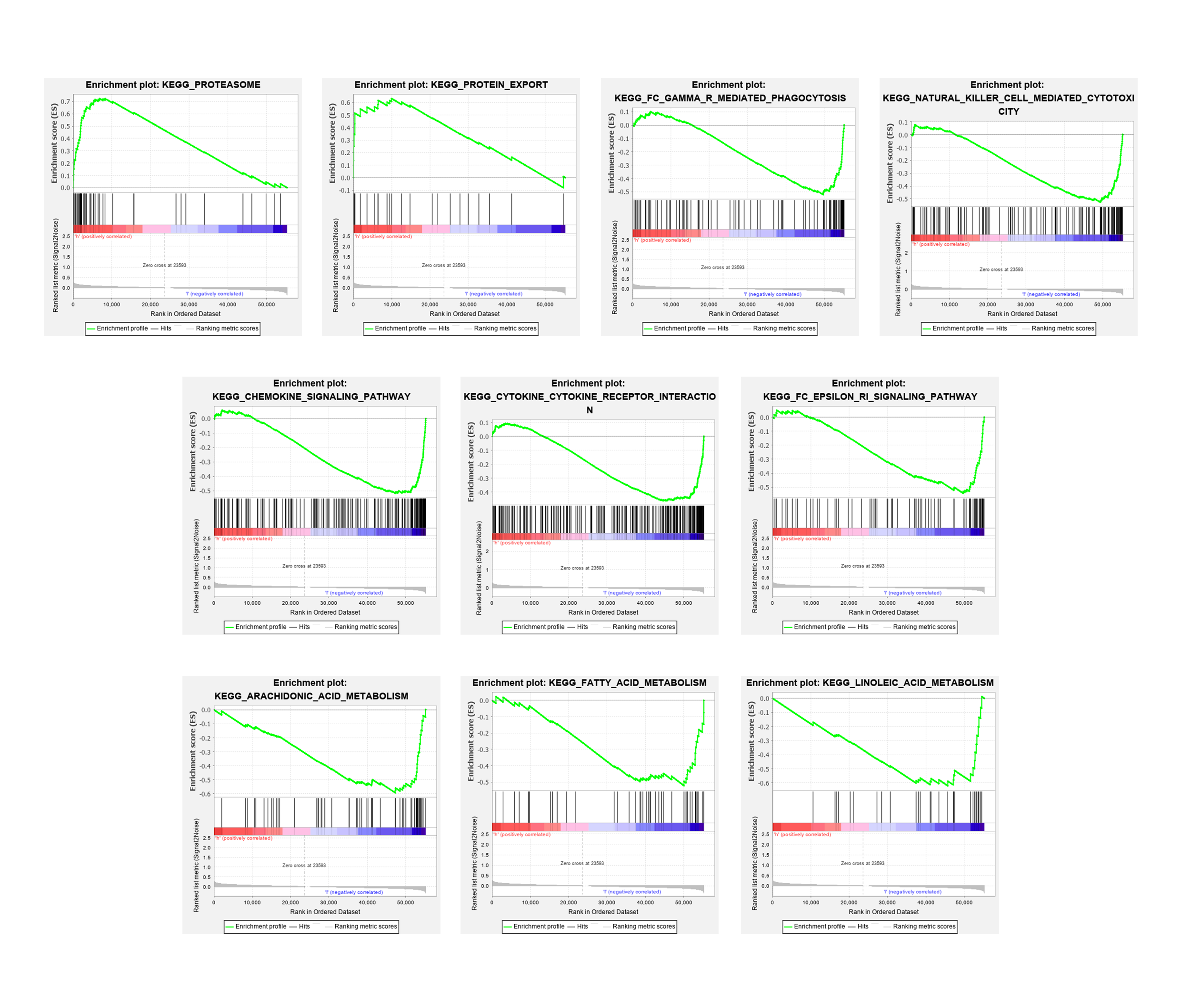

Supplement: Supplementary Figure 3 — Other single GSEA figures of the high-risk and low-risk groups. Single GSEA plots showing enriched pathways in the high-risk and low-risk groups displayed in Table 3, except the B cell receptor signaling pathway and T cell receptor signaling pathway. [file Image_3.TIF]
